# Efficacy of nutritional interventions to lower circulating ceramides in young adults: FRUVEDomic pilot study

**DOI:** 10.14814/phy2.13329

**Published:** 2017-07-12

**Authors:** Alice T. Mathews, Oluremi A. Famodu, Melissa D. Olfert, Pamela J. Murray, Christopher F. Cuff, Marianne T. Downes, Norman J. Haughey, Sarah E. Colby, Paul D. Chantler, I. Mark Olfert, Joseph W. McFadden

**Affiliations:** ^1^ Division of Animal and Nutritional Sciences West Virginia University Morgantown West Virginia; ^2^ West Virginia Clinical and Translational Science Institute Robert C. Byrd Health Sciences Center Morgantown West Virginia; ^3^ Department of Pediatrics West Virginia University School of Medicine Morgantown West Virginia; ^4^ Department of Microbiology, Immunology, and Cell Biology West Virginia University School of Medicine Morgantown West Virginia; ^5^ Division of Medical Laboratory Sciences West Virginia University School of Medicine Morgantown West Virginia; ^6^ Department of Neurology Johns Hopkins University School of Medicine Baltimore Maryland; ^7^ Department of Nutrition Science University of Tennessee Knoxville Tennessee; ^8^ Department of Exercise Physiology West Virginia University School of Medicine Morgantown West Virginia

**Keywords:** Ceramide, fruit and vegetable, insulin resistance, metabolic syndrome

## Abstract

The 2010 USDA Dietary Guidelines for Americans (DGA) recommends a diet largely composed of fruit and vegetables. Consuming a diet high in fruit and vegetables and low in refined carbohydrates and saturated fat may reduce an individual's risk for type 2 diabetes, nonalcoholic fatty liver disease, low‐grade chronic inflammation, and metabolic syndrome (MetS). Several recent studies have implicated the bioactive sphingolipid ceramide as an associative and causative biomarker for the development of these conditions. Considering that the intake of fruit and vegetables is frequently inadequate in young adults, we performed a pilot investigation to assess the efficacy of a free‐living fruit and vegetable intervention on overall metabolic health, circulating ceramide supply, and inflammatory status in young adults. We discovered that adoption of the recommended DGA for fruit and vegetable intake for 8 weeks decreased waist circumference, systolic blood pressure, and circulating cholesterol. Lipidomics analysis revealed that nutritional intervention can lower circulating ceramides, including C24:0 ceramide, a known inhibitor of insulin signaling. Unexpectedly, we observed an increase in C16:0 ceramide, suggesting that this form of ceramide in circulation is not associated with metabolic disease in humans. We also observed an improved inflammatory status with enhanced fruit and vegetable intake that was correlated with ceramide concentrations. These data suggest that adopting the recommended DGA is associated with a reduction of many, but not all, ceramide species and may help to prevent or mitigate MetS. Future research needs to assess whether the ceramide‐lowering ability of nutritional intervention is associated with reduced risk of developing metabolic disease.

## Introduction

Inadequate intake of fruit and vegetables represents an unhealthy dietary behavior that results in disproportionally higher rates of obesity‐related morbidity and mortality in young adults residing in the United States (Ford and Mokdad 2001; Field et al. 2003). Recent evidence has demonstrated that the inadequate consumption of fruit and vegetables can predispose individuals to an increased risk of developing the metabolic syndrome (MetS), type 2 diabetes mellitus, and cardiovascular disease (Serdula et al. 1996; Williams et al. 1999; Ford and Mokdad 2001; Lin and Morrison 2003). This elevated risk for metabolic disease is alarming if we consider that 78% of young adults consume less than the recommend amount of fruit and vegetables per day (McCracken et al. 2007). Epidemiological evidence indicates that the consumption of a diet enriched with fruit and vegetables, or one that is low in refined carbohydrates or saturated fats, can improve insulin sensitivity and lower circulating LDL‐cholesterol, and may provide a simple approach to lower an individual's risk of developing chronic metabolic dysfunction (Joshipura et al. 2001; Wang et al. 2005; Esmaillzadeh et al. 2006; Salas‐Salvadó et al. 2011). Given the clear link between inadequate nutritional intake and obesity‐related diseases, evaluating the effectiveness of dietary interventions to lower metabolic risk factors is necessary.

An important mechanism that regulates the progression of insulin resistance and related metabolic disorders involves the enhanced synthesis of the bioactive sphingolipid ceramide. Findings from in vivo investigations have implicated ceramides in the development of the MetS in association with diabetic dyslipidemia, hepatic lipid deposition, and inflammation, as reviewed by Holland and Summers (2008). Available evidence suggests that circulating ceramide regulates insulin signaling by preventing the translocation of glucose transporters to the plasma membrane through inactivation of PKB (Boon et al. 2013). In addition to antagonizing insulin signaling, elevations of lignoceroyl (C24:0) ceramide in lipoproteins have been demonstrated to increase levels of the inflammatory cytokine IL‐6 and monocyte chemoattractant protein‐1 (MCP‐1), and to decrease anti‐inflammatory IL‐10 expression in macrophages (Boon et al. 2013). Lowering ceramide concentrations by inhibiting synthesis improves insulin resistance, reduces hepatic lipid accumulation, and reduces inflammation associated with excess energy intake (Bikman and Summers 2011; Chavez et al. 2014). Based on these data, ceramides have been recognized as potential biomarkers for insulin resistance, nonalcoholic fatty liver disease (NAFLD), and MetS (Lopez et al. 2013; Meikle et al. 2013), however the potential for dietary recommendations to lower ceramide levels in humans has been underexplored.

Diets high in saturated fat increase hepatic ceramide synthesis and accumulation in rodents with impaired mitochondrial fatty acid processing and insulin sensitivity (Summers 2006; Chavez et al. 2014), conditions that often develop in parallel with NAFLD (Fabbrini et al. 2009). Specifically, palmitic acid is partitioned away from oxidation and transformed into alternative metabolic fates including ceramide. Although much of the data demonstrating the associative or causative relationship between ceramide and insulin resistance has been derived from high‐saturated fat‐feeding studies, Vilà et al. (2008) have established that hepatic ceramides are increased in rats fed fructose‐sweetened water. Vila and coworkers (2008) propose that the ability of fructose to promote ceramide synthesis may be attributed to impaired fatty acid *β*‐oxidation. Indeed, diets rich in refined carbohydrates such as those found in soft drinks can exacerbate NAFLD (Assy et al. 2008; Asrih and Jornayvaz 2014) and therefore may potentially increase hepatic ceramide synthesis and export.

Studies evaluating the efficacy of practical nutritional interventions to lower circulating ceramide in free‐living subjects are needed. We hypothesize that a dietary intervention focused on increasing the intake of fruit and vegetables, and reducing the consumption of refined carbohydrates or fat in free‐living subjects would lower serum ceramide concentrations in young adults. Our pilot investigation evaluated individuals that were between 18 and 28 years of age. Although, we employed a lipidomic approach, we focused our attention on palmitoyl (C16:0) and C24:0 ceramide because of their associative and causative role in the development of metabolic disease (Boon et al. 2013; Hla and Kolesnick 2014; Raichur et al. 2014; Turpin et al. 2014).

## Methods

The West Virginia University Institutional Review Board approved this research project (Clinical Trial Record 1409433435) that was initiated in January 2015. All participants provided written informed consent.

### Subject screening

To evaluate eligibility for participation in the study, young adults in the surrounding area of West Virginia University (Morgantown, WV) were screened via phone by a registered dietitian nutritionist (RDN). Subjects were asked a series of questions in five categories: (1) demographics (age, willingness to follow nutritional guidelines, height, weight, nationality, and pregnancy status), (2) metabolic risk factors (blood pressure, blood glucose, body shape (apple, pear, or no distinctive shape), medications taken, and family history of disease), (3) current nutritional status (meal frequency, food choices, and level of alcohol consumption), (4) physical activity level (frequency and type of physically activity), and (5) psychological and behavioral (satisfaction with weight and body shape, and assessment of risk for development of an eating disorder, and smoking/tobacco use). Individuals were excluded from the study if they were <18 years of age, pregnant, exhibited an eating disorder, treated for drug or alcohol abuse within the past year, received psychiatric treatment within the previous year, had used antibiotics in the last 3 months, displayed a body mass index (BMI) of less than 18, or weighed less than 50 kg.

To determine the risk for developing MetS, we developed a tool (called the Metabolic Syndrome Risk Score) to categorize individuals into “high”, “medium” or “low” risk of developing the MetS. The MetS risk score included the five components of the MetS (waist circumference, blood pressure, fasting glucose, and circulating triacylglycerol (TAG) and HDL‐cholesterol), but also considered nutritional status, physical activity, ethnicity, family history, and current medication use.

### Intervention design

Following subject screening, thirty‐six young adults (mean = 23.2 ± 2.5 years of age, range = 18–28 years of age) participated in the 8‐week free‐living nutritional intervention using principles of behavior change based on the Social Cognitive Theory. Although most individuals were randomly balanced into three dietary intervention groups, subjects residing together were assigned into the same dietary intervention to maximize compliance and minimize difficulties in adherence to the intervention by subjects living in the same household. The dietary interventions were based on the United States Department of Agriculture (USDA) MyPlate Dietary Guidelines for Americans (DGA; USDHHS, 2005) and were defined as follows: 50% of their meal plate must consist of fruit and vegetables (FRUVED; equivalent to 2.5 or 3 cups of vegetables for women and men, respectively, and 2 cups of fruit daily for all individuals). All subjects participated in the FRUVED diet, however, this intervention was divided into three groups: FRUVED (*n* = 12), FRUVED plus low refined carbohydrates (FRUVED + LRC; *n* = 12), or FRUVED plus low fat (FRUVED + LF; *n* = 12). Subjects that were allocated to the FRUVED + LRC intervention were counseled to consume low‐glycemic index foods (e.g., reduced intake of white bread, crackers, cake, and sugar‐sweetened beverages) and less than 3 oz. of refined carbohydrates, similar to reported guidelines (USDHHS, 2005). For FRUVED + LF, subjects were advised to consume a low‐fat diet, as recommended by the American Heart Association guidelines (Krauss et al. 2000). Subjects were instructed to consume 30% or less of caloric intake from total fat (equally divided into saturated, monounsaturated, and polyunsaturated fat), however the overall message conveyed was to choose low‐fat, and lean sources of dairy and protein in accordance with the USDA DGA.

### Education and monitoring

Enrolled subjects were required to attend a 2‐h group class (one for each of the different diets) by a RDN just prior to the start of the intervention. Subjects were provided instruction in basic nutrition guidelines, nutrition facts label reading, grocery shopping and budgeting for fruit and vegetable consumption, resources from local markets, as well as allowed and prohibited foods for their intervention. Materials were personalized for each specific diet to include sample menus and snack suggestions. All subjects were provided a culinary toolkit to secure success in fruit and vegetable preparation and planning of meals. The kit included knives, cutting board, peeler, can opener, measuring cups for dry and liquid ingredients, and plastic bags and containers for food storage. With the exception of diet, subjects were instructed to maintain pre‐intervention lifestyle behaviors, including physical activity and sleep patterns.

On a weekly basis, participants met with the RDN to be counseled on their intervention plan. The RDN examined dietary behaviors from weekly participant food logs, meal photos, and analysis of a 24 h dietary recall using Nutritionist Pro software (version 1.2, Nutritionist Pro, San Bruno, CA). By reviewing these measurements weekly, the RDN was able to counsel subjects on ways to further adhere to their assigned intervention while ensuring compliance. Apart from these assessments, the RDN and participant independently rated participant overall progress and adherence of assigned diet intervention on a continuum of 1–100 each week. This scale (which considered physical activity, stress, sleep, and medication use) was averaged weekly starting at week 2, and used to determine overall compliance at the end of the study. Upon completion of the study, if the average was below 90, individuals were excluded from analysis due to noncompliance to intervention. Monetary incentives were provided each week (following the completion of scheduled visitations with the RDN) to help off‐set the potential and/or perception of greater food expense of routinely purchasing fresh fruit and vegetables, and to help with compliance.

### Blood sampling and anthropometric measurements

Fasting blood was collected by venipuncture prior to the intervention, and at week 2, 5, and 8 of intervention. Blood was allowed to coagulate at room temperature for 30 min and then centrifuged at 3400*g* for 10 min at 4°C. Following centrifugation, serum was decanted and stored at −80°C until analysis. The West Virginia University, Ruby Memorial Hospital Clinical laboratory provided basic chemistry analysis (i.e., electrolytes, fasting blood glucose, alkaline phosphate, aspartate, and alanine aminotransferase (AST and ALT, respectively)), lipid panel (i.e., TAG, cholesterol, HDL, LDL, VLDL), and selected endocrine analyses (i.e., insulin, high‐sensitive C‐reactive protein) on blood samples collected pre (week 0) and post (week 8) intervention. Weekly physical activity was measured subjectively with physical activity logs and the International Physical Activity Questionnaire (IPAQ) pre and postintervention. Physical activity was also measured objectively with accelerometers (GT3X; ActiGraph, Pensicola, FL) during the first and last week. Anthropometric measurements were taken before and after intervention. Body fat was measured via BodPod (CosMed USA Inc., Chicago, IL), height by an electronic Stadiometer (SECA 213), body mass via digital scale (SECA 874), and waist, hip, and neck circumferences measured by Gulick tape. All measurements were taken twice and averaged by trained study personnel.

### Cytokine measurements

Circulating cytokine concentrations were measured from venous serum samples, using a Mesoscale V‐PLEX human pro‐inflammatory panel 1 (#K15049G; MesoScale Diagnostics, LLC., Gaithersburg, MD) and chemokine panel 1 (#K15047G) assay kits according to manufacturer protocol on a SECTOR Imager 2400 instrument (MesoScale Diagnostics, LLC.). Each subject's sample was assayed in duplicate and then averaged for final analysis. The average coefficient of variation of the standard curves generated compared to the expected standard values was less than 10% for all measured cytokines. Minimum detection levels for cytokines were as follows: IL‐6 1.31 pg/mL, IL‐10 0.59 pg/mL, INF‐*γ* 4.09 pg/mL, TNF‐*α* 0.87 pg/mL, MCP‐1 0.06 pg/mL, MCP‐4 1.51 pg/mL, macrophage‐derived chemokine (MDC) 2.11 pg/mL, thymus‐ and activation‐regulated chemokine (TARC) 0.14 pg/mL, and eotaxin 1.70 pg/mL.

### Ceramide measurements

Serum ceramide extraction was conducted, using previously established methods that employ a modified Bligh and Dyer procedure including C12:0 ceramide as an internal standard (Avanti Polar Lipids, Alabaster, AL; Haughey et al. 2004; Rico et al. 2015). The organic layers from plasma extracts were dried in a nitrogen evaporator (Organomation Associates Inc., Berlin, MA) and resuspended in pure methanol prior to analysis. Ceramides were detected by multiple reaction monitoring using liquid chromatography coupled with electrospray ionization tandem mass spectrometry (ESI/MS/MS; API3000; AB Sciex Inc., Thornhill, ON, Canada) operated in positive mode. Liquid chromatography and MS/MS parameters have been previously described (Mielke et al. 2015). Slight differences in extraction efficiency and fluctuations in mass spectrometer efficiency were normalized to the C12:0‐ceramide internal standard. Instrument efficiency was monitored daily and individual plasma extracts were reanalyzed if the internal standard deviated more than 20% from the overall median internal standard value. The original or rerun data with internal standard concentrations closest to the median C12:0‐ceramide value were included in the final analysis. Plasma ceramide concentrations were determined by fitting the identified ceramide species to standard curves based on acyl‐chain length. Ceramide standards were purchased from Sigma‐Aldrich (C16:0, C18:0, and C18:1; St. Louis, MO) or Avanti Polar Lipids (C20:0, C22:0, C24:0, and C24:1). Glycosylated ceramides were purchased from Matreya Inc. (C24:0‐GlcCer, C16:0‐LacCer, C18:0‐LacCer; Pleasant Gap, PA). Instrument control and quantitation were performed, using Analyst 1.4.2 and MultiQuant software (AB Sciex Inc.), respectively.

### Statistical analysis

An analysis of variance (ANOVA) was used to examine difference among the groups. Anthropometric and plasma data were analyzed as repeated measures over time relative to start of intervention under the MIXED procedure of SAS (version 9.3; SAS Institute Inc.). The statistical model included the random effect of subject, and the fixed effects of intervention and week relative to start of intervention, and their interaction. The model included post hoc adjustment for multiple comparisons, using Tukey's adjustment. The method of Kenward–Rogers was used for calculation of denominator degrees of freedom. Normality of the residuals was evaluated with normal probability, box plots, and homogeneity of variances with plots of residual versus predicted values. When necessary, data were transformed but presented in the original scale. When the intervention effect or intervention × week interaction was significant, the SLICE option of SAS was used to compare intervention differences at individual time points. Studentized residual values >3.0 or <−3.0 were considered outliers and removed from the analysis (typically 1 per response variable). Parametric Pearson correlations were performed in order to determine associations between plasma ceramides, anthropometric measurements, and circulating cytokines. All results are expressed as least squares means and their standard errors, unless stated otherwise. Significance was declared at *P* ≤ 0.05, and trends toward significance were considered at 0.05 <*P* ≤0.10.

## Results and Discussion

The principal finding of our study is that young adults who are overweight (BMI between 25 and 30) and “at‐risk” of developing MetS show improved metabolic health following adoption of the USDA recommended DGA of consuming 50% fruit and vegetables, as evidenced by reductions in circulating ceramide, biomarkers of inflammation, systolic blood pressure, and waist circumference. It is important to emphasize the current intervention was a “free‐living” study design permitting subjects to freely choose the type and amount of food they consumed within their own environment with the requirement that their meals meet the recommended 50% fruit and vegetable consumption. While this approach does not control total nutritional intake, the purpose of this study was not to induce weight loss per se, but rather determine if adopting the DGA for fruit and vegetables would produce measureable clinical changes and/or biomarkers linked with the progression of cardiometabolic disease.

Although thirty‐six subjects participated, six subjects were not included within our final analyses because the average weekly compliance score fell below the compliance threshold. Therefore, our data only report the data from thirty compliant subjects with a mean MetS risk score of 4.1 ± 0.3 and a mean age of 23.6 ± 0.4 (range = 18 to 28 years of age; 53% female; 66% Caucasian; Table 1). The moderate MetS risk score was primarily attributed to subjects being overweight (BMI = 27.2 ± 5.9; Table 2), as subjects displayed normal concentrations of circulating fasting triglycerides, glucose and HDL‐cholesterol, as well as normal systolic and diastolic blood pressure (Table 2 and 3). At study, enrollment, demographics, and MetS risk score were comparable for the FRUVED, FRUVED + LRC, and FRUVED + LF interventions; however, the spread of ethnicity was lower in the FRUVED group.

**Table 1:** Metabolic syndrome (MetS) risk score and demographics for compliant intervention subjects.^a^

	All subjects *n* = 30	FRUVED *n* = 12	FRUVED + LRC *n* = 8	FRUVED + LF *n* = 10
MetS risk score^b^	4.1 ± 0.3	4.4 ± 0.5	4.0 ± 0.6	3.8 ± 0.5
Age, years	23.6 ± 0.4	23.7 ± 0.4	22.1 ± 1.2	24.8 ± 0.6
Female	16 (53)	7 (58)	5 (62)	6 (60)
Male	14 (47)	5 (42)	3 (38)	4 (40)
White	20 (66)	10 (83)	3 (38)	7 (70)
African American	2 (7)	0	2 (25)	0
Hispanic	3 (10)	0	2 (25)	1 (10)
Asian	3 (10)	1 (8)	1 (12)	1 (10)
Other	2 (7)	1 (8)	0	1 (10)
Appalachian	18 (80)	9 (75)	4 (50)	5 (50)
Non‐Appalachian	12 (40)	3 (25)	4 (50)	5 (50)

a Dietary interventions based on the United States Department of Agriculture MyPlate Dietary Guidelines for Americans included fruit and vegetables (FRUVED), FRUVED plus low refined carbohydrates (FRUVED + LRC), or FRUVED plus low fat (FRUVED + LF). Values within parenthesis represent percent of *n*.

b Cumulative score determined by the five components of the MetS (waist circumference, blood pressure, fasting glucose, and circulating triacylglycerol and HDL‐cholesterol), as well as nutritional status, physical activity, ethnicity, family history, and current medication use. Scores ranged from 1 to 7 on a 12‐point scale. Increased scores are indicative of increased risk for developing the MetS.

**Table 2:** Anthropometric and blood pressure measurements before and after intervention.^a^

	All subjects (*N* = 30)		SEM	*P*‐value		
	Pre	Post		Group	Time	Group × Time
Weight, kg	77.0	76.0	14.8	0.49	0.07	0.48
BMI, kg/m^b^	26.4	26.0	4.2	0.33	0.05	0.88
Body fat, %	28.6	25.9	12.5	0.60	0.14	0.37
Waist circumference, cm	83.4	80.6	11.1	0.76	<0.001	0.54
Hip circumference, cm	102.9	102.2	9.3	0.36	0.14	0.36
Neck circumference, cm	36.0	35.6	3.7	0.97	0.01	0.47
Systolic pressure, mm/Hg	118.6	111.8	12.5	0.39	<0.01	0.57
Diastolic pressure, mm/Hg	62.7	60.3	8.3	0.36	0.12	0.76

a Dietary interventions based on the United States Department of Agriculture MyPlate Dietary Guidelines for Americans included fruit and vegetables (FRUVED; *n* = 12), FRUVED plus low refined carbohydrates (FRUVED + LRC; *n* = 8), or FRUVED plus low fat (FRUVED + LF; *n* = 10). Values are presented as least squared means ± SEM average of pre (week 0) and post (week 8) intervention.

b Body mass index.

**Table 3:** Serum metabolic health measurements before and after intervention.^a^

	All subjects (*N* = 30)		SEM	*P*‐value		
	Pre	Post		Group	Time	Group × Time
Glucose, mg/dL	86.9	87.1	8.4	0.29	0.87	0.17
Triglycerides, mg/dL	92.7	87.9	31.2	0.77	0.24	0.23
Cholesterol, mg/dL	181.0	171.7	29.2	0.55	0.05	0.67
HDL‐cholesterol, mg/dL	56.6	50.3	11.4	0.96	<0.001	0.50
LDL‐cholesterol, mg/dL	105.7	103.7	23.4	0.42	0.66	0.41
VLDL‐cholesterol, mg/dL	18.6	17.7	1.2	0.76	0.25	0.21
AST, U/L^b^	25.4	22.7	2.4	0.42	0.31	0.09
ALT, U/L^c^	28.7	25.4	5.3	0.31	0.24	0.81
Insulin, *μ*IU/mL	7.0	7.4	3.2	0.18	0.82	0.12
HOMA‐IR^d^	1.5	1.6	0.8	0.10	0.82	0.08
Na, mmol/L	139.1	138.2	2.4	0.30	0.10	0.57
K, mmol/L	3.8	4.0	0.3	0.72	<0.01	0.86

a Dietary interventions based on the United States Department of Agriculture MyPlate Dietary Guidelines for Americans included fruit and vegetables (FRUVED; *n* = 12), FRUVED plus low refined carbohydrates (FRUVED + LRC; *n* = 8), or FRUVED plus low fat (FRUVED + LF; *n* = 10). Values are presented as least squared means ± SEM average of pre (week 0) and post (week 8) intervention.

b Aspartate aminotransferase.

c Alanine aminotransferase.

d Homeostasis model assessment‐estimated insulin resistance.

### Nutrient intake

Prior to intervention, the subject included in the analysis consumed less than the recommend 5 cups/d of fruit and vegetables (by the 2010 USDA DGA; Table 4). Following the 8‐week intervention, all subjects exceeded recommendations by consuming 108% more fruit and vegetables (*P* < 0.001), compared to pre‐enrollment. Moreover, intake of total empty calories (defined as calories from added sugar and added fat by DGA) and fat was reduced in all subjects by 56% and 8%, respectively (*P* < 0.05). Regarding fat intake, the consumption of saturated fat was reduced by 8.3 ± 1.5 g postintervention (*P* < 0.05), whereas intake of unsaturated fat or cholesterol was not modified. In contrast, total fiber and sugar intake increased by 74% and 34%, respectively (*P* < 0.05). Specifically, all subjects consumed more insoluble and soluble fiber as well as several sugars including glucose, fructose, and sucrose, relative to pre‐intervention. Only a few differences in the measured dietary components were seen among the three dietary arms. Specifically, FRUVED + LRC consumed less soluble and insoluble fiber, relative to FRUVED + LF (*P* < 0.001). Collectively, the data suggests that the FRUVED + LRC or FRUVED + LF did not modify intake of refined carbohydrates or fat, relative to FRUVED. The reason for no observed differences could be a limitation of using self‐reported measurements of dietary intake or that our pilot study did not include a nonintervention control group. Alternatively, consuming more fruit and vegetables may concomitantly decrease fat and processed carbohydrate intake.

**Table 4:** Daily intervention intake assessment.^a^

	All subjects (*N* = 30)		SEM	*P*‐value		
	Pre	Post		Group	Time	Group × Time
Total calories, Kcal	2263.3	1935.7	498.8	0.96	0.17	0.59
Empty calories, Kcal^b^	997.6	441.6	594.2	0.54	<0.001	0.87
Fruit and vegetables, cups	2.5	5.2	2.2	0.70	<0.001	0.51
Total carbohydrates, g	228.3	259.6	20.3	0.81	0.17	0.64
Total fiber, g	19.2	34.0	13.9	0.12	<0.001	0.95
Insoluble fiber, g	1.4	3.3	2.3	<0.001	<0.01	0.71
Soluble fiber, g	0.3	0.8	0.5	<0.01	<0.001	0.82
Total sugar, g	78.7	105.4	38.8	0.96	0.03	0.78
Glucose, g	7.8	15.3	1.6	0.24	<0.001	0.31
Fructose, g	8.5	16.8	1.7	0.57	<0.001	0.30
Galactose, g	0.1	1.1	0.3	0.44	0.05	0.96
Sucrose, g	10.1	14.2	2.1	0.13	<0.01	0.79
Lactose, g	6.2	6.3	1.9	0.14	0.31	0.86
Maltose, g	0.5	0.7	0.2	0.23	0.01	0.94
Total protein, g	90.9	91.4	7.9	0.58	0.92	0.39
Total fat, g	90.1	64.7	8.4	0.84	<0.01	0.36
Saturated fat, g	28.8	20.5	17.4	0.60	0.04	0.20
Monounsaturated fat, g	21.0	16.8	17.5	0.59	0.73	0.19
Polyunsaturated fat, g	13.9	10.1	10.9	0.44	0.67	0.60
Cholesterol, mg	227.3	247.4	463.1	0.18	0.98	0.67

a Dietary interventions based on the United States Department of Agriculture MyPlate Dietary Guidelines for Americans included fruit and vegetables (FRUVED; *n* = 12), FRUVED plus low refined carbohydrates (FRUVED + LRC; *n* = 8), or FRUVED plus low fat (FRUVED + LF; *n* = 10). Values are presented as least squared means ± SEM average of pre (week 0) and post (week 8) intervention.

b Added calories from fat and sugar.

### Assessment of metabolic health

Although subjects were clinically healthy at the start of intervention, we observed a significant decrease in waist circumference and systolic blood pressure in all subjects by week 8 (3% and 6%, respectively; *P* < 0.01; Table 2). We did not observe changes in body weight, BMI, body fat %, hip and neck circumference, or diastolic blood pressure following intervention (Table 2). Epidemiological studies have demonstrated lower BMI with higher fruit and vegetable intake (Serdula et al. 1996; Williams et al. 1999; Lin and Morrison 2003), however a decrease in adiposity is not always associated with this feeding behavior (Field et al. 2003). Contrasting anthropometric conclusions may be attributed to differences in total energy intake, duration of behavior, demographics, type of food, or method of food preparation. Because waist circumference and systolic blood pressure are strong risk factors for the development of MetS, type 2 diabetes and cardiovascular disease (Srinivasan et al. 1996; Wang et al. 2005), we consider the observed decreases in these measurements to be favorable intervention outcomes. Comparable to our anthropometric evaluation, we did not observe changes in glucose, insulin, estimated insulin sensitivity, LDL‐cholesterol, VLDL‐cholesterol, AST, or ALT (Table 3). However, we did detect lower circulating cholesterol by week 8 (5%; *P* = 0.05). In contrast, we observed a significant decrease in HDL‐cholesterol (*P* < 0.001) postintervention, where higher levels are associated with atheroprotective properties (Rader and Hoving 2014) and others have observed a similar HDL‐cholesterol response following a high fiber intervention (Jenkins et al. 1993). To positively impact HDL‐cholesterol, researchers suggest replacing saturated fat with unsaturated fat instead of decreasing overall fat intake (Muller et al. 2003), which was not a component of our study design.

Previous work has demonstrated an inverse relationship between the incidence of diabetes and the consumption of fruit and vegetables (Ford and Mokdad 2001), a response that may be attributed to increased intake of dietary fiber, and decreased consumption of fat and refined carbohydrates as observed in our investigation. Additionally, our subjects were instructed not to change physical activity levels during the study in order to prevent confounding beneficial influences that could be conferred by exercise. Given the physical activity levels were found to not be different between pre versus postmeasures in all our subjects, we believe the improved metabolic health was due solely to change in nutrition.

### Modifications in circulating ceramides

Current evidence suggests that the development of insulin resistance, NAFLD, and MetS is intrinsically linked with the accumulation of ceramide (Holland et al. 2007; Bikman and Summers 2011; Brozinick et al. 2012). Increased caloric intake from saturated fat can activate hepatic ceramide synthesis in overweight subjects (Haus et al. 2009; Holland et al. 2011), and subsequently increase lipoprotein ceramide packaging and export (Boon et al. 2013). In turn, circulating lipoprotein ceramide can mediate inflammatory cytokine release from adipose tissue macrophages, and inhibits insulin‐stimulated PKB activation in skeletal muscle (Boon et al. 2013). These proposed mechanisms are consistent with data from several studies that have reported elevated circulating ceramide in obese adolescents, adults with type 2 diabetes, or rodents with NAFLD (Haus et al. 2009; Lopez et al. 2013; Kasumov et al. 2015). Avoiding body weight gain, or consuming diets low in saturated fatty acids or fructose from nonfruit sources are a likely means to increase hepatic fatty acid oxidation, inhibit ceramide synthesis, and lower circulating ceramide to improve metabolic health (Dubé et al. 2011; Dekker et al. 2013). To assess this possibility, we employed a lipidomic approach to quantify an array of serum nonglycosylated and glycosylated ceramides in young adults consuming a diet enriched with fruit and vegetables.

**Figure 1:**
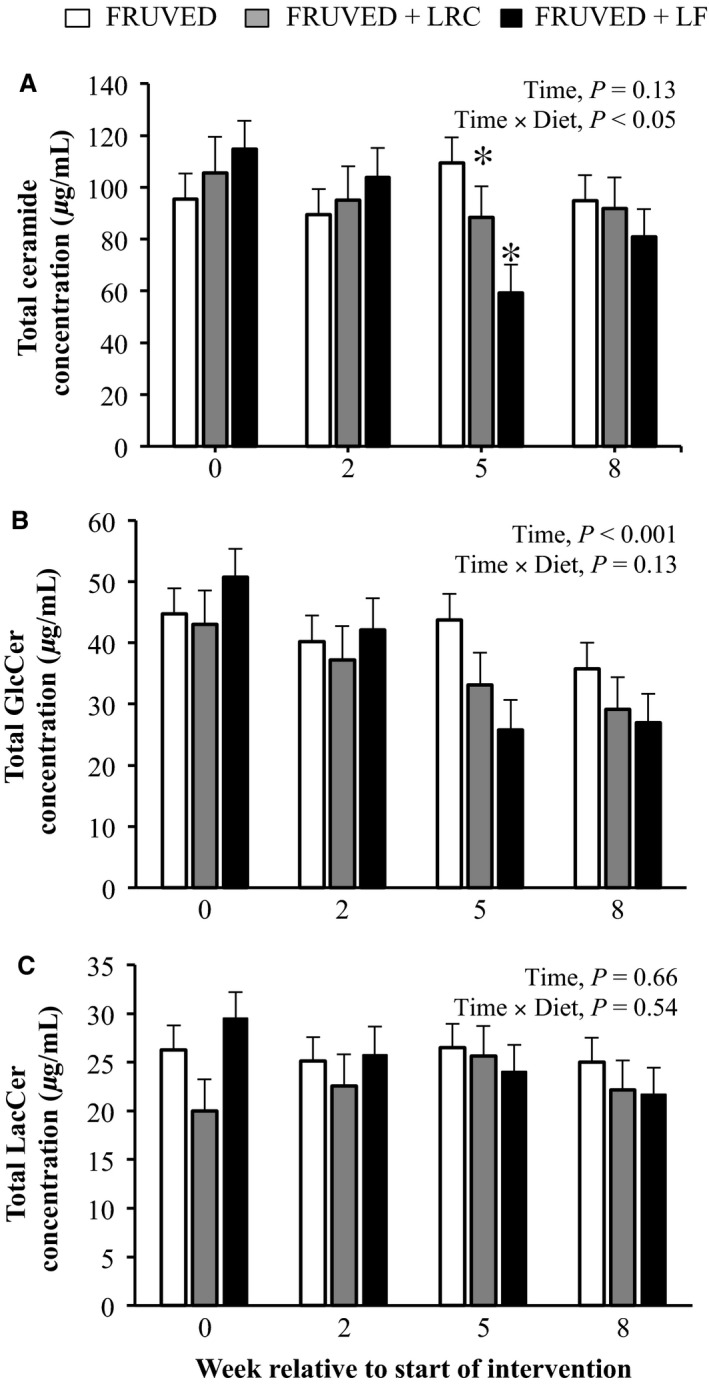
Total (A) ceramide, (B) GlcCer, and (C) LacCer concentrations in FRUVED (50% fruit and vegetable), FRUVED + LRC (FRUVED plus low refined carbohydrate), and FRUVED + LF (FRUVED plus low fat) groups throughout intervention. Data are represented as least squares means and their standard errors. Statistical significance compared to FRUVED group within time point. *P < 0.05.

**Figure 2:**
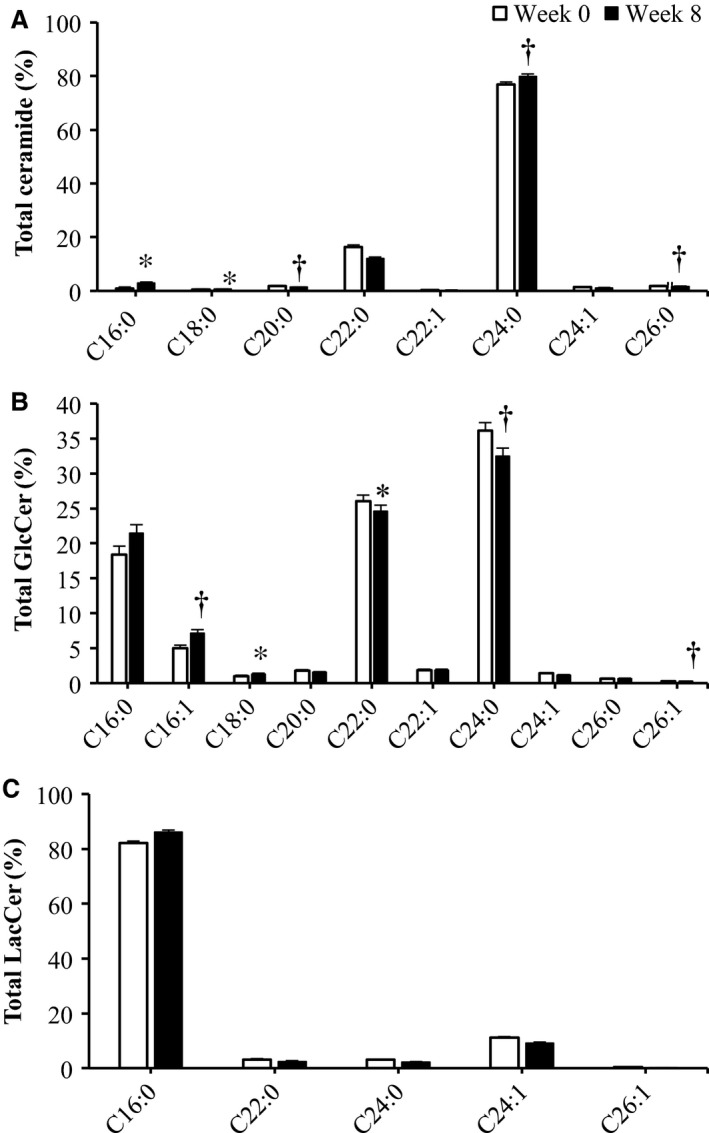
Abundance profile of (A) Ceramide, (B) GlcCer, and (C) LacCer in all subjects. Dihydro species are not included. Data are represented as least squares means and their standard errors. Statistical significance compared to pre‐intervention (week 0) measurements within acyl moiety. *P < 0.05; ^†^ *P* < 0.10.

The intake of a diet high in fruit and vegetables but low in refined carbohydrates and fat decreased circulating total ceramide concentrations by week 5 in young adults (16% and 48% for FRUVED + LRC and FRUVED + LF, respectively; *P* < 0.05; Fig. 1). However, we did not observe a change in total ceramide levels in the FRUVED group. Previous research has confirmed that C24:0 ceramide is the most abundant ceramide in circulation (Haus et al. 2009), a subspecies produced by hepatic ceramide synthase 2 (Laviad et al. 2008) and exported within liver‐derived lipoproteins (Lightle et al. 2003; Boon et al. 2013). Indeed, we observed that C24:0 ceramide was highly abundant in serum, representing 77% of total ceramide at the initiation of intervention (Fig. 2). Relative to C24:0 ceramide, we detected low circulating levels of C16:0 ceramide (0.9% of total ceramide) which is synthesized by ceramide synthase 6 (Turpin et al. 2014).

**Figure 3:**
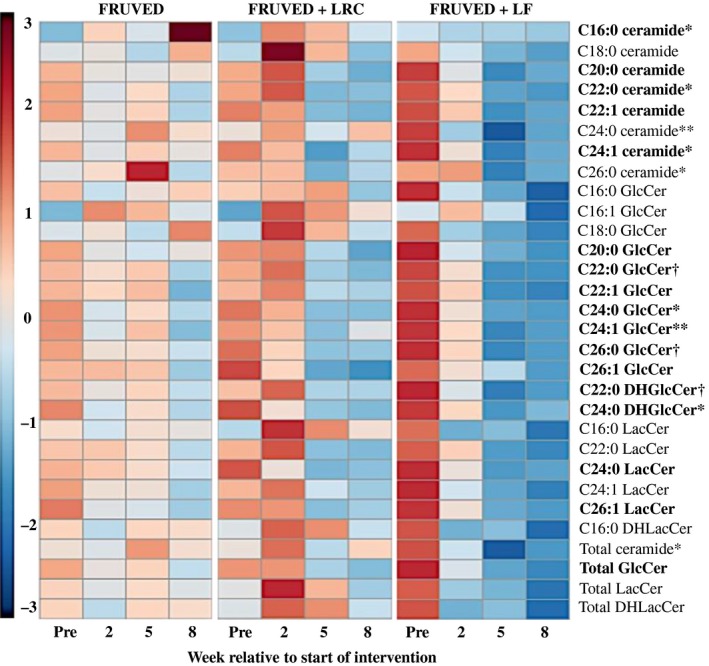
Heat map representation of ceramide and glycosylated ceramide changes with intervention. Fruit and vegetable interventions (FRUVED) with or without low intake of refined carbohydrates (LRC) and fat (LF) are shown. Heat map represents the magnitude of mean‐centered, fold change increases (red) or decreases (blue) for each sphingolipid relative to samples pre‐intervention (week 0). Comparisons should be made within metabolite and intervention. Heat map was generated using MetaboAnalyst 3.0 (Xia et al. 2012). Intervention × time effects are presented as **P < 0.01, *P < 0.05, and ^†^ *P* < 0.10. Significant time effects (*P* < 0.05) are presented in bold.

**Figure 4:**
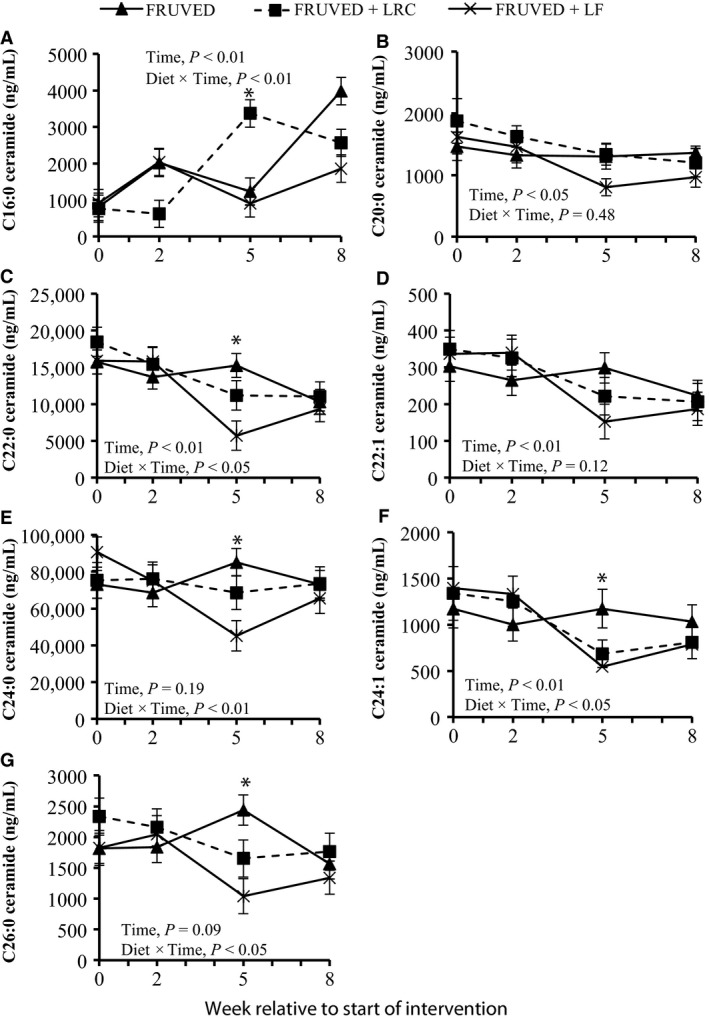
Changes in circulating ceramides during intervention. Interventions include FRUVED (50% fruit and vegetable), FRUVED + LRC (FRUVED plus low refined carbohydrate), and FRUVED + LF (FRUVED plus low fat). Statistical significance compared to FRUVED group within time point. Statistical significance compared to FRUVED group within time point. *P < 0.05.

We discovered that the majority of circulating ceramides decreased with time for FRUVED + LRC and FRUVED + LF interventions (Fig. 3); albeit the response was often greater for FRUVED + LF. For instance, the concentrations of C22:0, C24:1, and C26:0 ceramide were significantly lower by week 5 of intervention (29% to 64% for FRUVED + LRC and FRUVED + LF; *P* < 0.05; Figs. 3 and 4); ceramide moieties associated with increased BMI and fasting blood glucose in adolescents with type 2 diabetes (Lopez et al. 2013). Most notably, subjects following a diet containing high fruit and vegetables with low dietary fat displayed a 50% decrease in C24:0 ceramide by week 5, relative to the start of intervention (*P* < 0.01; Fig. 4). We view the lowering of C24:0 ceramide concentration in serum as a favorable intervention outcome because lipoprotein C24:0 ceramide inhibits PKB activation and glucose transporter translocation in skeletal muscle (Boon et al. 2013), and circulating C24:0 ceramide is inversely related to insulin‐stimulated glucose disposal in humans (Meikle et al. 2013). High dietary intake of saturated palmitic acid can increase C24:0 ceramide accrual (Larsen and Tennagels 2014). Although we did not detect a change in total or saturated fat intake for FRUVED + LF, relative to FRUVED, the FRUVED + LF group may have exhibited a lower intake of palmitic acid, a fatty acid required for de novo ceramide synthesis. Alternatively, hepatic palmityl‐CoA supply may have been limited in these subjects because of suppressed de novo lipogenesis or enhanced mitochondrial oxidation. These possibilities may also explain the ceramide‐lowering ability of FRUVED + LRC. Moreover, differences in circulating and liver fatty acid pools may explain why FRUVED alone was unable to lower ceramide.

**Figure 5:**
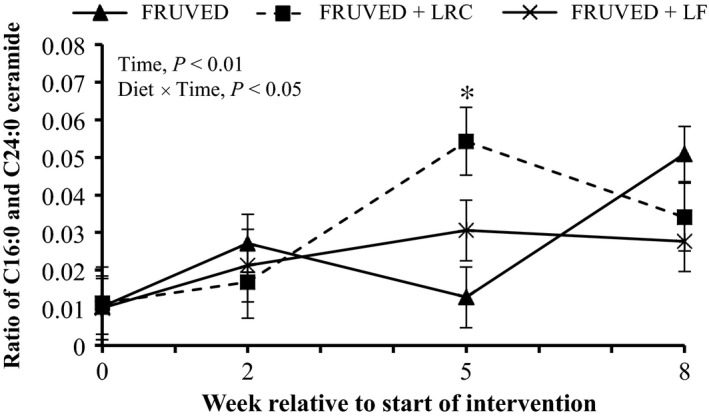
Ratio of C16:0 to C24:0 ceramide in serum. Interventions include FRUVED (50% fruit and vegetable), FRUVED + LRC (FRUVED plus low refined carbohydrate), and FRUVED + LF (FRUVED plus low fat). Statistical significance compared to FRUVED group within time point. Statistical significance compared to FRUVED group within time point. *P < 0.05.

Although the majority of circulating ceramides declined following intervention, we did observe an 80%, 70%, and 50% increase in circulating C16:0 ceramide by week 8 for FRUVED, FRUVED + LRC, and FRUVED + LF, respectively (*P* < 0.05; Fig. 4). Furthermore, the ratio of C16:0 to C24:0 ceramide increased 66% across time (*P* < 0.01; Fig. 5). We and others have previously observed a reciprocal relationship between circulating C16:0 and C24:0 ceramide (Raichur et al. 2014; Rico et al. 2017). Researchers that have recently evaluated tissue ceramide supply and insulin action have proposed that C16:0 ceramide is a proapoptotic mediator of the pathophysiology of insulin resistance in response to a high saturated fat diet, and in contrast, C24:0 ceramide confers antiapoptotic and proliferative functions (Raichur et al. 2014; Turpin et al. 2014). Nevertheless, our measured increase in serum C16:0 ceramide that developed concurrently with lower circulating cholesterol and reduced waist circumference is a paradoxical observation. In support of our findings, Meikle et al. (2013) did not observe a relationship between circulating C16:0 ceramide and type 2 diabetes. The ability of C16:0 ceramide to antagonize insulin action may be reliant upon the intracellular tissue concentration of this sphingolipid which was not characterized in our investigation. Additionally, the ability of extracellular lipoprotein ceramide to antagonize insulin signaling may involve a different mode of action since long‐chain ceramides are highly hydrophobic and cannot pass freely through the plasma membrane (Goñi and Alonso 2006). The relationship between the acyl chain length of circulating ceramide and insulin‐stimulated glucose disposal requires further investigation to definitively distinguish C16:0 or C24:0 ceramide as the causal agent of insulin resistance in humans.

### Modifications in circulating glycosylated ceramides

We also measured serum GlcCer, a glycosphingolipid formed via glycosylation of ceramide by the enzyme glucosylceramide synthase. We observed that serum total GlcCer concentrations were lower for all subjects by week 8 (20%, 32%, and 46% for FRUVED, FRUVED + LRC, and FRUVED + LF, respectively; *P* < 0.001; Fig. 1), relative to start of interventions. Similar to nonglycosylated ceramides, GlcCer levels are elevated in overweight animals (Chavez et al. 2014; Rico et al. 2015); however, the ability of circulating GlcCer to directly antagonize insulin resistance remains uncertain. To further evaluate glycosphingolipid biology in our subjects, we quantified serum LacCer that are formed by a (1‐4)‐*β* linkage of galactose to GlcCer. In our investigation, serum total LacCer levels were not modified by intervention (Fig. 1). Recent evidence suggests that nonglycosylated ceramides are stronger circulating biomarkers for diabetes or prediabetes in adults as compared with GlcCer or LacCer (Meikle et al. 2013). Nevertheless, both nonglycosylated ceramides and GlcCer have been implicated in the pathogenesis of insulin resistance at the cellular level (Chavez et al. 2014).

**Figure 6:**
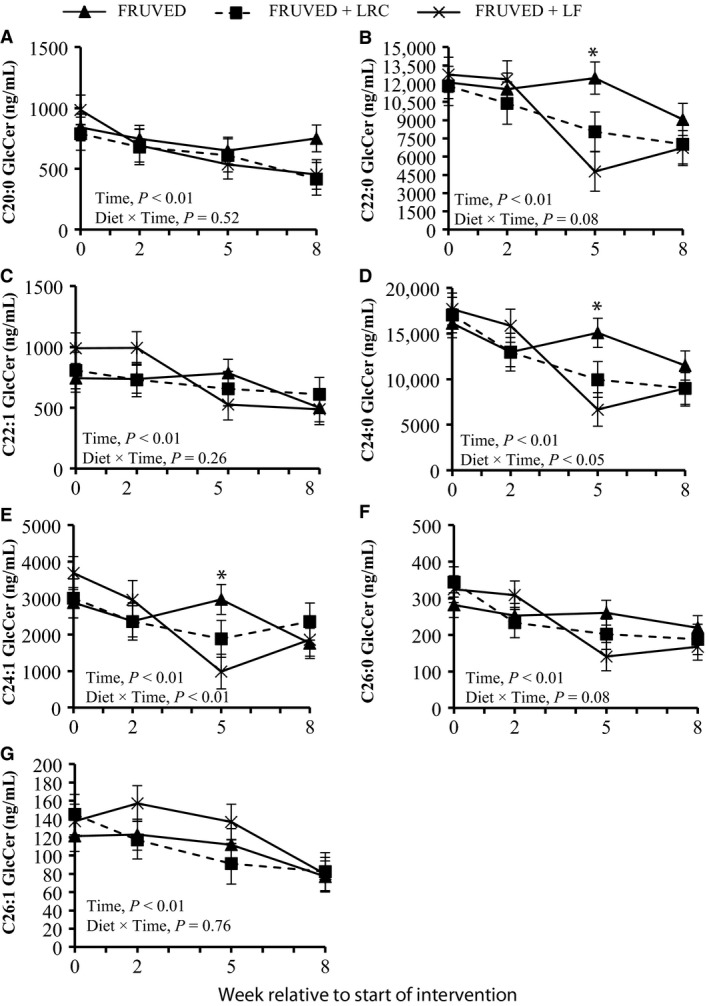
Changes in circulating monohexosylceramides (GlcCer) during intervention. Interventions include FRUVED (50% fruit and vegetable), FRUVED + LRC (FRUVED plus low refined carbohydrate), and FRUVED + LF (FRUVED plus low fat). Statistical significance compared to FRUVED group within time point. *P < 0.05.

**Figure 7:**
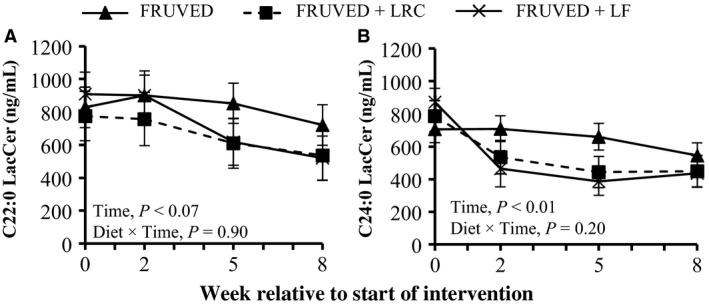
Changes in circulating lactosylceramides (LacCer) during intervention. Interventions include FRUVED (50% fruit and vegetable), FRUVED + LRC (FRUVED plus low refined carbohydrate), and FRUVED + LF (FRUVED plus low‐fat) compared to FRUVED group.

**Figure 8:**
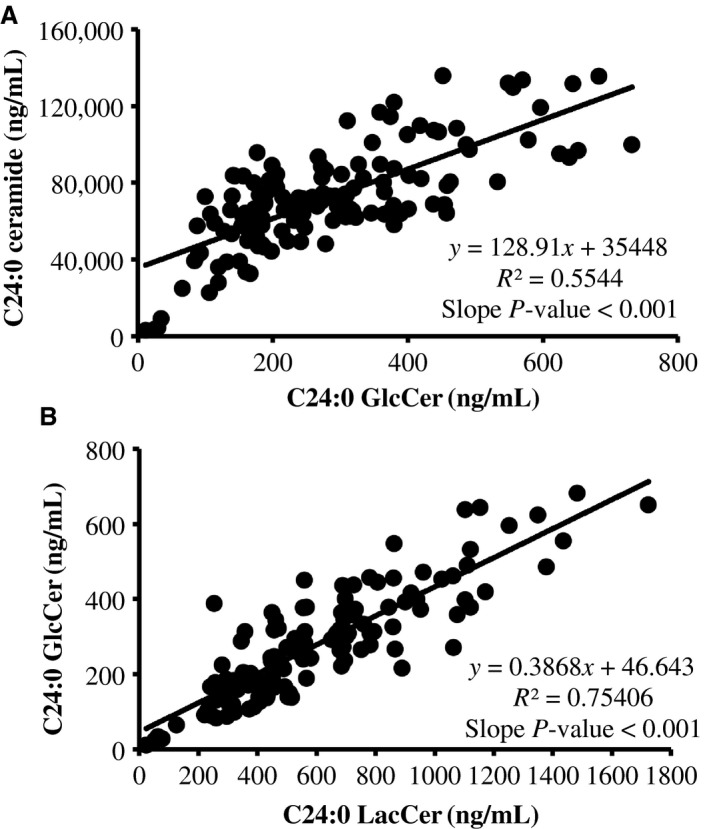
Random regression analysis between serum concentrations of (A) C24:0 ceramide and monohexosylceramide (GlcCer), and (B) C24:0 GlcCer and lactosylceramide (LacCer). Coefficients of determination (R2) represent the association between observed and model predicted values. Data represent measurements for week 0, 2, 5, and 8 relative to the start of interventions.

Specifically, we observed that C24:0 GlcCer and C16:0 LacCer were the most abundant species in each lipid subclass (Fig. 2). Moreover, changes in serum GlcCer were similar to the supply of ceramide (Figs. 3 and 6). Relative to the start of each intervention, we observed a 31–73% decrease in serum C22:0, C24:0, and C24:1 GlcCer by week 5 in FRUVED + LRC and FRUVED + LF subjects (*P* < 0.05), respectively. In contrast, intervention did not modify serum C16:0, C20:0, and C26:1 GlcCer levels. For LacCer sphingolipids, we detected a decrease in C22:0 and C24:0 LacCer with time (17% and 37% decrease by week 5; *P* < 0.01; Figs. 3 and 7); however, we did not observe changes in the concentrations of serum C16:0, C24:1, or C26:1 LacCer with intervention. Furthermore, we observed a strong positive association between C24:0 ceramide and GlcCer, and between C24:0 GlcCer and LacCer across all sampled subjects (Fig. 8). These results may indicate a coordinated regulation of synthesis for glycosphingolipids with the C24:0 acyl moiety. Although glycosylated ceramides may mediate the link between obesity and insulin resistance (Aerts et al. 2007), the causal role of glycosphingolipids in the progression of insulin resistance is actively being defined. Current evidence suggests that the insulin receptor is co‐localized with glycosphingolipids in plasma membrane caveolae (Vainio et al. 2002), and excessive ganglioside content can disrupt insulin signaling by displacing the insulin receptor from these microdomains (Kabayama et al. 2004). Modifying dietary energy intake is a likely means to control glycosylated ceramide supply. For instance, mice fed a low fat diet exhibit lower circulating C20:0, C22:0, and C24:0 GlcCer levels as compared with mice fed a high fat diet (Barber et al. 2012). It is important to recognize that the observed ceramide and GlcCer‐lowering response associated with increased fruit and vegetable consumptions developed even though dietary fructose intake increased. Therefore, the ability of dietary fructose to augment ceramide synthesis likely depends on compromised hepatic function which was not evident in our investigation as demonstrated by clinically low concentrations of circulating triacylglycerol, VLDL‐cholesterol, AST, and ALT, as supported by Vila and coworkers (2008).

### Inflammation and ceramides

Consuming a diet rich in fiber and monounsaturated fat while reducing intake of saturated fat and cholesterol can increase fat expenditure and reduce low‐grade chronic inflammation (Esposito et al. 2003), therefore we assessed the inflammatory status of our subjects. Although we did not detect changes in circulating cytokines between our diet intervention groups, our nutritional intervention did lower circulating MCP‐4 and TARC in all subjects by week 8 (*P* < 0.05; Table 5). We also observed a tendency for serum anti‐inflammatory IL‐10 concentration to increase (*P* = 0.06); however no changes in IL‐6, TNF‐*α*, MCP‐1, eotaxin, or INF‐*γ* levels were observed. The ability of fruit and vegetable intake to suppress inflammation may be due to bioactive nutrients found in these foods including folate, flavonoids, and antioxidants (Watzl et al. 2005; García‐Lafuente et al. 2009). Specifically, the consumption of fruit or vegetables, or the intake of vitamin C and *β*‐carotene is inversely associated with pro‐inflammatory markers in adolescents (Holt et al. 2009).

**Table 5:** Serum cytokine measurements before and after intervention.^a^

	All subjects (*N* = 30)		SEM	*P*‐value		
	Pre	Post		Group	Time	Group × Time
IL‐6	4.1	4.5	0.5	0.59	0.39	0.99
IL‐10	2.1	2.4	0.5	0.84	0.06	0.84
IFN‐*γ*	23.5	19.7	4.4	0.24	0.84	0.93
TNF‐*α*	10.5	10.3	0.6	0.51	0.19	0.46
MCP‐1^b^	245.7	207.2	31.5	0.52	0.26	0.54
MCP‐4	152.3	89.7	26.2	0.44	0.02	0.11
MDC^c^	1299.7	1176.6	139.1	0.38	0.08	0.36
TARC^d^	222.9	167.0	37.5	0.31	0.04	0.71
Eotaxin	126.6	99.9	18.7	0.86	0.34	0.30

a Dietary interventions based on the United States Department of Agriculture MyPlate Dietary Guidelines for Americans included fruit and vegetables (FRUVED; *n* = 12), FRUVED plus low refined carbohydrates (FRUVED + LRC; *n* = 8), or FRUVED plus low fat (FRUVED + LF; *n* = 10). Values are presented as least squared means ± SEM average of pre (week 0) and post (week 8) intervention.

b MCP, monocyte chemoattractant protein.

c MDC, macrophage‐derived chemokine.

d TARC, thymus‐ and activation‐regulated chemokine.

The ability of inflammation to increase the synthesis of ceramide has been documented (Holland et al. 2011; Boon et al. 2013). For instance, saturated fatty acids can activate proinflammatory receptor toll‐like receptor 4 which increases the expression of serine palmitoyltransferase and ceramide synthase in skeletal muscle, and activate hepatic sphingomyelinase (Holland et al. 2011). In our study, multiple ceramides were positively correlated with pro‐inflammatory cytokines (Table 6). For example, C20:0, C22:0, and C22:1 ceramide were positively associated with MDC, TARC, TNF‐*α*, and eotaxin (*r* = 0.33–0.54; *P* < 0.05). A similar relationship was observed for a limited number of GlcCer or LacCer subspecies. Although the majority of sphingolipids measured were not correlated with INF‐*γ* or IL‐10, highly abundant C24:0 ceramide and GlcCer were significantly correlated with serum INF‐*γ* and IL‐10 (*r* = 0.34 and −0.35; *P* = 0.05), respectively. Interestingly, the inhibition of ceramide synthesis can negate the antagonistic effects of inflammation on insulin sensitivity (Holland et al. 2011), therefore dietary interventions designed to lower circulating ceramide may be a means to improve insulin sensitivity in overweight subjects experiencing inflammation.

**Table 6:** Correlations between cytokines and ceramides.^a^

	IL‐6		IL‐10		IFN‐*γ*		TNF‐*α*		MCP‐1^b^		MCP‐4		MDC^c^		TARC^d^		Eotaxin	
	*r*	*P*	*r*	*P*	*r*	*P*	*r*	*P*	*r*	*P*	*r*	*P*	*r*	*P*	*r*	*P*	*r*	*P*
Ceramide																		
C16:0	−0.03	0.85	−0.03	0.85	0.06	0.73	−0.11	0.53	−0.05	0.77	−0.04	0.83	0.01	0.91	−0.08	0.67	−0.01	0.93
C20:0	0.29	0.10	−0.09	0.62	0.05	0.78	0.33	0.07	0.09	0.61	−0.03	0.83	0.36	0.04	0.34	0.05	0.40	0.02
C22:0	0.27	0.13	−0.20	0.26	0.17	0.34	0.34	0.06	0.16	0.38	−0.07	0.69	0.36	0.04	0.40	0.02	0.42	0.01
C22:1	0.26	0.15	−0.14	0.43	0.28	0.13	0.41	0.02	−0.02	0.89	−0.11	0.55	0.43	0.01	0.51	0.01	0.54	0.01
C24:0	0.21	0.25	−0.01	0.97	0.34	0.05	0.29	0.11	0.19	0.31	−0.03	0.85	0.29	0.11	0.26	0.15	0.27	0.13
C24:1	0.06	0.72	−0.28	0.13	0.08	0.64	0.39	0.02	−0.09	0.61	−0.22	0.23	0.26	0.16	0.35	0.05	0.36	0.04
GlcCer																		
C20:0	0.29	0.10	−0.27	0.14	0.09	0.62	0.31	0.08	0.15	0.41	0.01	0.94	0.31	0.09	0.29	0.11	0.40	0.02
C22:0	0.27	0.13	−0.29	0.11	0.27	0.13	0.27	0.14	0.29	0.11	0.02	0.91	0.26	0.15	0.30	0.09	0.37	0.04
C24:0	0.21	0.25	−0.35	0.05	0.28	0.12	0.25	0.16	0.29	0.11	−0.01	0.93	0.20	0.28	0.29	0.11	0.36	0.05
LacCer																		
C22:0	0.32	0.08	−0.25	0.18	0.12	0.51	0.29	0.10	0.06	0.72	−0.12	0.49	0.34	0.06	0.32	0.07	0.41	0.02
C24:0	0.15	0.40	−0.40	0.02	−0.01	0.99	0.27	0.14	0.02	0.89	−0.05	0.76	0.26	0.15	0.24	0.18	0.32	0.07

a Pearson's correlation between ceramides and inflammatory cytokines. Data are representative of samples collected at week 0 and 8 relative to the start of intervention.

b MCP, monocyte chemoattractant protein.

c MDC, macrophage‐derived chemokine.

d TARC, thymus‐ and activation‐regulated chemokine.

## Conclusion

In summary, our pilot study demonstrates that an 8‐week free‐living fruit and vegetable intervention designed according to the USDA MyPlate guidelines can lower waist circumference, reduce systolic blood pressure, and decrease circulating cholesterol in young adults. Moreover, we establish that a short‐term nutritional intervention can lower ceramide, a biomarker for metabolic disease. Of particular interest, we observed lower levels of circulating C24:0 ceramide with intervention which is a known antagonist of insulin action. Although the majority of ceramides were lowered by the study intervention, we unexpectedly observed an increase in serum C16:0 ceramide raising the possibility that circulating C16:0 ceramide may not be a causal agent of insulin resistance in humans. In addition to the changes in ceramide supply, a fruit and vegetable intervention lowered circulating inflammatory cytokines. Collectively, these data suggest that consuming a healthy diet improves clinical indices and biomarkers of metabolic and cardiovascular health, irrespective of changes in body fat mass. Future work with a larger sample size is needed to better understand the effects of macro or micronutrients on specific species of ceramides, and the mechanisms by which these ceramides exert their action, as well as their etiology in the development of obesity and chronic diseases such as the MetS, diabetes and cardiovascular disease.

## Conflicts of Interest

No conflicts of interest, financial or otherwise, are declared by the authors.
